# Dr. L. P. Haskell, on Dental Prosthesis

**Published:** 1896-01

**Authors:** 


ARTICLE IV.

### DR. L. P. HASKELL, ON DENTAL PROSTHESIS.

**Delivered before the Washington State Dental Society, 1895.**

I congratulate the younger members of the profession in having commenced their practice in these days of diffusion of knowledge and greater liberality and esprit de corps in the profession than in the early days of cruder methods, when the secrets of the laboratories and offices were kept religiously guarded.

When I commenced dentistry plaster was just beginning to supplant beeswax for the taking of impressions, and I immediately adopted it and consider it the only reliable ingredient for that purpose. At that time tin was still used for the manufacture of dies, but after experimenting with type metal, platinum, zinc and tin, even having brass castings made in brass factories, I was induced to try Babbit's metal, and have continued to use it in the making of dies ever since. I found it was the only method that possessed the essential qualifications for dentist's dies: viz. sufficient hardness, toughness and smoothness. My formula for its manufacture is one part copper, two of antimony and one of tin. Some have found it serviceable to substitute silver in place of tin, but I consider the above formula the safest proportion, as more tin renders the dies too soft and with less they are too brittle and apt to crack.

I always make my metal plates fit the mouth closely, and never have any difficulty in making them adhere properly without an air chamber. I believe the use of the latter usually results in failure on account of subsequent changing in the process. In cases where the plate rocks over a hard palate I use a relief consisting of a thin film of wax.

I was practicing dentistry fourteen years before the introduction of vulcanized rubber. After using it some time I began to notice the changes produced by it in the absorption of the alveolar process, which I had never observed before its introduction. I attribute this change principally to the fact that rubber being a non-conductor causes undue retention of heat, resulting in a constant change in the process; while others attribute it to the fact that rubber prevents the formation of new tissue.

In a visit to Boston after a long absence, I had occasion to examine some very old cases of mine where I had used gold plates, and having been in use for forty years, and found no change in the process whatever; while in about five years' use of vulcanite very marked changes have taken place. I very much disapprove of the use of rubber for permanent work, particularly in full upper cases, where the air cannot circulate and there is considerable atmospheric pressure. However, in the case of the lower jaw the same objection does not hold good; and in many of such cases rubber proved to be the best thing that could be used. Since the introduction of aluminum I do not see any good reason for the use of rubber for permanent work and believing that patients could be made to see the false economy of ordering it, especially where, in many cases they are willing to pay the highest price for everything else they wear. Nothing improves the appearance so much as artificial teeth, to say nothing of the increased comfort and convenience; and I can only account for the purchase of a cheap denture in such cases from the fact that the plates, not being visible, the love of ostentation of such people was satisfied as long as the teeth—the only portion of the work visible—presented a respectable appearance.

The principal objection to the old aluminum extracted from clay was owing to the presence of iron, which caused it to disintegrate and become porous; but by reason of the new process of manufacturing aluminum by electricity that objection is removed. I find no apparent change in plates made from this metal, and they appear to wear as well as plates made from any other metal. It is a very beautiful metal to swage, and when used pretty thick makes a very stiff, unyielding plate; while under the old process of etching the rubber did not seem to adhere as well to the aluminum as to gold plates. All aluminum plates should be swaged, as I do not believe it is advisable to cast aluminum plates, owing to the fact that no practicable method of soldering it has been discovered; and it is impossible to reduce the fitting of the plates to an exact science, especially where allowance has to be made for shrinkage, alterations could be more readily made on swaged plates.

In response to a question by Dr. Andruss as to the effect of alkaline solution under heat upon aluminum, and as to whether it produced a metallic taste in the mouth, Dr. Haskell replied that the heat which could be taken into the mouth would not affect it, and that any metallic taste complained of in the mouth could be probably attributed to idiosyncrasy of the individual, as he had never found any greater complaints of that kind in his experience with aluminum than with platinum or other metals. I strongly advocate the use of platinum as a substitute for rubber. I make it a practice to induce patients, if possible, to have gold plates, and failing in that I recommend aluminum in preference to rubber.

In swaging any metal, whether gold or platinum, I always make it a practice to cut the plate, and thereby save considerable time in swaging, especially in cases of considerable undercut. I prefer to make a straight cut, instead of the old fashioned V shape, as I believe it strengthens the plate to lap the edges over and solder, and there is no objection to it. In case of very heavy undercuts, to avoid the tearing away of the counter-die and plate, I swage very thoroughly as far as possible, then burnish those portions of the plate not swaged, with a burnisher.

For the purpose of annealing I do not believe in the use of oil but prefer using a Bunsen Burner and rubbing the plate over with the butt end of a match until it produces a slight color, from charring.

In cases of very heavy undercuts, where it is impossible to mould, I overcome the difficulty by using double cores, or a core in two sections, of plaster and asbestos; which, being more fibrous, will hold together better than anything I have been able to discover for the purpose of mixing with plaster.

In lower cases, having a very thin alveolar ridge, to avoid breaking of the ridge, a little sand is pressed under the undercut, even though the counter-die may not fit the die, and then swage as far as it will go, then burnish to place—in this way I am able to get a perfect plate.

After long experience I find that I can fit the mouth better with metal than with rubber plates; the latter is apt to warp and contract across the heel of the plate during vulcanizing, which causes them to bear harder on any ridge there may be in the roof of the mouth. I do not believe in scraping at the posterior portion of the plate unless the tissues are soft on either side of the palatal ridge in the center, and then only in those soft places.

A peculiarity about the lower jaw is the fact that the teeth on the left side are higher and longer and fuller than on the right. In articulation the anterior teeth should be arranged symetrically regardless of the position of the others, for the reason that they give expression to the mouth, and in arranging the upper teeth it is often difficult to get the teeth on the left side far enough back, owing to the lower teeth coming up too high, this peculiarity is confined to the left side and is not the case on the right.

It is often the case that when the teeth are all extracted the left side of the lower jaw diverges further from the meridian line, and that could be overcome by setting the teeth farther in over the ridge on the left side than on the right. I believe there are more failures from faulty articulation in the case of dentures of full upper plates than from any other cause. The six anterior teeth should never be allowed to meet—a space should be left not only for present but for future changes when the teeth and jaw come close together in the course of time.

A patient should never be allowed to see or examine the denture until everything is fully articulated.

In the use of articulating paper I obtain the best results by getting the patient to bite rapidly up and down instead of grinding on the paper, and the articulation should be so arranged that the pressure will come on the bicuspids and the first molar. I have known of instances where the wisdom teeth or second molar of the lower jaw stood at an angle of 45 degrees, and in the articulation of an upper set those teeth should never be permitted to come together end for end as it will cause crowding of the plates forward. In case the lower teeth are far enough forward to permit it, I recommend the dropping of another tooth behind them from above in order to throw the crowding backwards instead of forwards. I believe articulation to be one of the most important branches of prosthetic dentistry and the most likely, if neglected, to produce an unfavorable impression on the patient by the teeth not coming together properly.

In case the plate is found to press on the posterior margin of the mouth I recommend the grinding of the articulation of the last molars rather than the filling or alteration of the plate.

In reply to a question of Dr. Price as to the proper course to pursue in order to make an upper denture satisfactory, where there were no lower posterior teeth remaining in the mouth, the Doctor replied, "I recommend putting some in as I consider it absurd to have a complete upper set with no lower posterior teeth as to have a two-wheeled vehicle with only one wheel, especially as the primary object in inserting artificial teeth is for the purpose of mastication."

In reply to another question as to the practicability of attaching a full bridge to the cuspid roots, he replied that in most cases where full dentures were attached to 2, 3 or 4 roots, I have always found them unsatisfactory.

In all cases of bridges and partial artificial dentures, the pressure of the articulation should be thrown on the natural teeth, because, if thrown on the crown or bridge it would have a tendency to crowd the plate into ths gums and cause additional absorption and discomfort.

In lower plate work, in case one good lower tooth remains in the mouth it should by all means be retained as affording a good anchorage, for the lower denture, no matter what tooth it is. I do not believe, however, that there is any use in retaining the superior cuspids if all the other teeth are gone, because a more successful denture could be inserted with a full plate than with a partial one of this kind. I believe dentists are very foolish in advising patients to retain those upper cuspids under the idea that they tend to preserve the contour of the lips and face. This I do not believe to be the fact, on the contrary I believe they are a source of infinite trouble to the patient and tend to weaken the upper plate.

In reply to a question by Dr. Wright, Dr. Haskell said: "In swageing any metal I always oil my dies to prevent, as far as possible, the baser metals adhering to the plate, and before annealing wipe off all trace of the baser metals. After annealing and partial swageing, wash the plates in sulphuric acid and boil them so as to peel off the base metals. I prefer the use of cotton-seed oil for mixing modeling sand, to that of water, the steam from which caused the formation of air bubbles in the metal cast."

In reply to a question by Dr. Holmes, Dr. Haskell said "In cases where the teeth are all gone on one side I would for the purpose of making a perfect clasp, advise the separation of the teeth, and if a considerable space is required I would, in order to prevent the induction of decay in the teeth, advise the extraction of one sound tooth, if necessary, to secure a firm hold for the artificial teeth. I believe this to be for the best interest of the patient in the end.

I would advise never to put wet borax on hot teeth as it is liable to crack the teeth. I greatly disapprove of the too general use of cross pins by dentists. In my practice I use the so called straight pins exclusively except in some special cases of bridge-work, as I believe they are much stronger and less liable to break. In chosing artificial teeth select bicuspids and molars with the long lingual cusps. For clasp metal I use gold alloyed with platinum, 16 to 1, or a pennyweight of platinum to an ounce of gold.

As a proof of the fact that nature works according to geometrical rule or law in the arrangement of the teeth, I wish to mention the discoveries of Dr. Bonwill in comparative anatomy, and the result of his investigations, covering a wide field and including examinations of many thousands of skulls, of pre-historic as well as modern races. Dr. Bonwill has discovered the law underlying the formation of the teeth to be that in all perfectly arranged jaws the six anterior teeth formed the arc of a circle, the radius of which circle could be determined by the width of the central, lateral and cuspid teeth it making no difference whether the teeth were wide or narrow. In any given case by taking the measurement of the central, lateral and cuspid teeth, and forming a circle of which that would be the radius, it will be found that a line drawn through the center of that circle would come through the center of the second bicuspid; a line drawn across the posterior margin of the second molar would coincide with a line drawn across the posterior periphery of that circle. The third molar does not seem to cut any figure in the economy of nature so far as the arrangement of the teeth is concerned. Dr. Bonwill's general law enables him, by having as a basis any single tooth in the upper jaw, to tell with certainty, from the width of that tooth what would be the corresponding width of every other tooth.

I have never found this rule to fail in any natural set of teeth; and acting upon that rule I always form a brass disk according to the measurements of the six anterior teeth, and adjust the articulation according to that circle. In the arrangement of artificial teeth I find it an excellent guide; the edge of the circle coming on the cutting edge of the six anterior teeth and also on the first bicuspids.

Dr. Bonwill's law does not apply as to artificial teeth in regard to the centre of that circle coming through the center of the second bicuspid; because in artificial teeth it was very rarely, if ever, that one could find a set of teeth where the bicuspids and molars were in proper proportion; and this has been a source of exceeding annoyance to me in the course of my practice. I often find a set of very wide incisors in connection with little, narrow, insignificant bicuspids and molars.

In arranging a set of teeth for an old person whose teeth have been extracted for many years, the processes are usually absorbed so that there is practically nothing left, and the lower jaw projects forward, its radius seeming to be shortened; therefore it is a very difficult matter to arrange the teeth in such cases, as there is no rule to go by and no basis to start from as to the exact position of the teeth.

In arranging the lower set to the upper set I always begin with the second bicuspid, arranging the fronts but partially, the main thing being to secure articulation of the bicuspid and molar, and then the anterior teeth can be articulated in the best way possible. Sometimes they will be found too wide for the space allotted them, and in that case they can be lapped or crowded in somehow. The main thing is to articulate the bicuspids and molars properly. The chief difficulty in articulating bicuspids and molars is owing to the fact that the articulating surfaces are not made to come together, it being necessary to grind some of them to make them meet. The lingual cusps of the bicuspids and molars should be shorter than the buccal cusps of the lower, and vice versa. I frequently find instances where the lingual cusps of the superior bicuspids or molars are longer than the buccal. I follow Dr. Bonwill's method of using the articulating circle by grinding deep, broad grooves in the bicuspids and molars so as to bring the surfaces together, and I believe that there is no other method that can be followed with satisfaction until it is possible to induce dental depots to furnish artificial teeth more in accordance with nature.—*Pacific Dental Journal.*

